# Case report: Artery of Percheron infarction as a rare complication during atrial fibrillation ablation

**DOI:** 10.3389/fcvm.2022.914123

**Published:** 2022-09-13

**Authors:** Xin Xie, Xuecheng Wang, Jinbo Yu, Xiaoqian Zhou, Liya Shi, Jian Zhou, Yizhang Wu, Zijun Chen, Baowei Zhang, Xiaorong Li, Bing Yang

**Affiliations:** Department of Cardiology, School of Medicine, Shanghai East Hospital, Tongji University, Shanghai, China

**Keywords:** artery of Percheron, atrial fibrillation, catheter ablation, complication, stroke

## Abstract

The incidence of stroke or transient ischemic attacks (TIA) in atrial fibrillation (AF) catheter ablation procedures is around 1% and may be unnoted under anesthesia. The artery of Percheron (AOP) infarction is a rare kind of stroke with heterogeneity in manifestation, which further makes the perioperative early detection and diagnosis a challenge. Herein, we present one patient who underwent AF ablation and presented mental status alteration after withdrawing anesthetics. An emergency head CT was obtained, which revealed no apparent pathological changes. A late MRI test confirmed the diagnosis of AOP infarction. With oral anticoagulants and rehabilitation therapies, the patient’s awareness improved and fully recovered on the sixth-month follow-up. Variability in manifestation, no positive radiological finding on initial CT, and a low incidence has made few clinicians to gain much experience with this type of infarct, which delays the diagnosis and initiation of appropriate treatment.

## Introduction

Atrial fibrillation (AF) is the most common sustained cardiac arrhythmia in adults, and catheter ablation has been recommended as the first-line therapy (1). With the development of devices and a deeper understanding of AF, the ablation procedure has proven to be a safe operation, especially in high-volume centers (2). However, though presenting a low incidence, severe complications such as death, atrio-esophageal fistula, tamponade, and stroke or transient ischemic attacks (TIA) still happen in clinical practice (3, 4). Early detection and treatment play important roles in the management of these complications.

Thalami are symmetric, paired, midline structures of the diencephalon regulating consciousness, sleep, and alertness (5). The thalamus and the midbrain have a complex arterial supply mainly originating from the posterior cerebral artery (PCA) (6). The artery of Percheron is one of four variants of this complex blood supply system and only counts 0.7% of all (5, 7). The artery of Percheron (AOP) infarction usually happens with cardioembolism and small artery disease (5, 8), presenting a set of symptoms, such as bilateral vertical gaze palsy, memory impairment, coma, hypersomnolence, akinetic mutism, and behavioral disorders (8, 9). Its heterogeneity and complexity in symptoms often delay the diagnosis of this rare stroke. As late diagnosis often happens, seldom could thrombolytic therapy be performed within the therapeutic window, which may further worsen the prognosis.

We report a case of a patient who underwent AF catheter ablation under local anesthesia and presented severe drowsiness after withdrawing anesthetics. The MRI scan taken 3 days later revealed a bilateral infarction of the thalamus, confirming the diagnosis of AOP infarction. A well understanding of this unusual procedure complication may further improve the management.

## Case description

**FIGURE 1:**
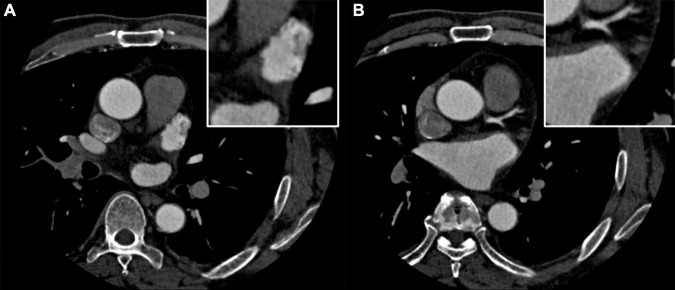
**(A,B)** Preoperative left atrium computed tomography (CT) angiography excludes the intracardiac thrombus.

A 58-year-old man was admitted to the cardiovascular department due to palpitation and shortness of breath. He was diagnosed with paroxysmal atrial fibrillation 3 years ago, and no treatment is given. Recent ECG and Holter monitoring confirmed its persistent episode. After being anticoagulated (rivaroxaban 20 mg daily) for 3 weeks, he was admitted and prepared for AF catheter ablation. He had a history of well-controlled hypertension. Physical examination and ECG confirmed the diagnosis of persistent AF. All blood tests and echocardiography were within normal limits, and preoperative left atrium CT angiography demonstrated no intracardiac thrombus (Figures 1A,B).

Radiofrequency catheter ablation was performed under local anesthesia with intravenous fentanyl and dexmedetomidine. The detailed periprocedural management and ablation strategy has been described previously (10). In brief, the patient underwent pulmonary vein isolation first and cardioversion was used to restore sinus rhythm. Detailed voltage mapping and drug provocation revealed a low voltage zone and premature atrial contractions at the left atrial posterior wall. Accordingly, substrate modification was performed. Intravenous heparin was given to maintain an activated clotting time (ACT) of 300 ± 50 s during the procedure. The vitals were stable during the procedure, and no sinus arrest or pericardial effusion was noted.

However, after withdrawal of all anesthetics, the patient remains severe drowsiness. The vitals were stable and physical examination showed that the pupils were equal and reactive. On the neurological examination, the patient presented a fluctuant consciousness state fully reversible under pain stimuli and bilateral positive pathologic signs. After having a consultation with the neurology and anesthesiology department, lasting effects of anesthetics and acute cerebral infarction were highly suspected. Naloxone was prescribed but no symptom relief was noted after intravenous drip. Then, an emergency head CT was obtained 2 h after the ablation procedure, and it revealed no apparent pathological changes. He was admitted to the cardiac care unit for further observation.

**FIGURE 2:**
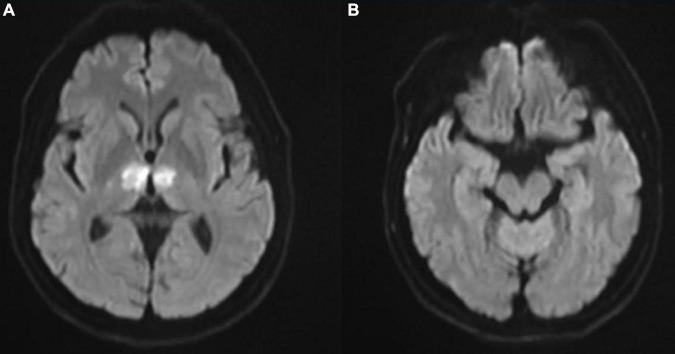
**(A)** Axial diffusion-weighted magnetic resonance imaging (MRI) showing bilateral paramedian thalamic hyperintense signal due to restricted diffusion and **(B)** no involvement on the midbrain level.

**FIGURE 3:**
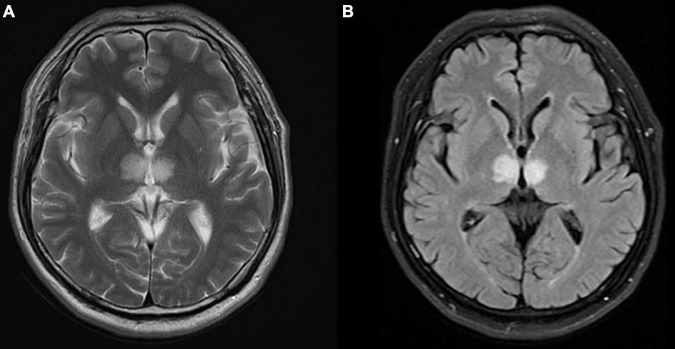
Axial magnetic resonance imaging (MRI) presenting bilateral high-signal intensity on the paramedian thalamus in **(A)** T2-weighted sequence, and **(B)** FLAIR sequence.

In the cardiac care unit, the patient’s consciousness gradually improved but still presented moderate drowsiness. To ascertain the pathogenesis of altered consciousness, a head MRI was ordered on the third day after the procedure. It showed symmetrical lesions in the paramedian thalamus, in the territory of the artery of Percheron. The lesions presented with an abnormal restriction of water diffusion on diffusion-weighted imaging (DWI) and hyperintensity in FLAIR and T2-weighted sequence on the paramedian thalamus level, without the midbrain involved (Figures 2, 3). MRI and corresponding clinic manifestations confirmed the diagnosis of an AOP infarction.

Routine oral anticoagulants and supporting therapies were prescribed after the procedure. Due to the late diagnosis of AOP infarction, thrombolytic therapy was not applied, and rehabilitation therapy was soon prescribed after MRI. By the fifth day after the procedure, the patient’s consciousness gradually recovered and presented slight dyscalculia and memory impairment. Computed tomography perfusion was done but still revealed no abnormalities. On the tenth day, the patient was discharged with outpatient follow-up. After following up for 6 months, the patient has fully recovered without any neurological deficiency. A timeline of events is given in Supplementary Figure 1.

## Discussion

Atrial fibrillation is the most common sustained cardiac arrhythmia in adults around the world, counting for 2–4% of the population (11). Catheter ablation has been recommended as the first-line therapy, especially for patients who are refractory to drugs or present with heart failure (1). The incidence of symptomatic stroke and TIA was reported around 1% (4, 12). The mechanism of embolism formation during the procedure includes air emboli in sheaths or was generated by heating blood, particulate emboli originating from tissue denaturation, and thromboses due to activation of the coagulation system (13). In the case of this patient, the ACT was kept for 300 ± 50 s during the procedure, and bubbles were not noticed by either catheter movement or X-ray. Tissue denaturation might be the most reasonable explanation for stroke. However, as thrombus aspiration was not performed, the definite reason for stroke still remained unclear. Most cerebral embolisms during the procedure are asymptomatic, and impaired left ventricular ejection fraction and low ACT levels may be associated with episodes (14). As reported in our case, the patient underwent a symptomatic AOP infarction during ablation, which has not been reported before. Normal preoperative echocardiography and a compliance ACT level during the procedure had proven for the patient to be away from mentioned risk factors, while a higher ACT control level (>350 s) might further reduce the embolic risk (15).

**FIGURE 4:**
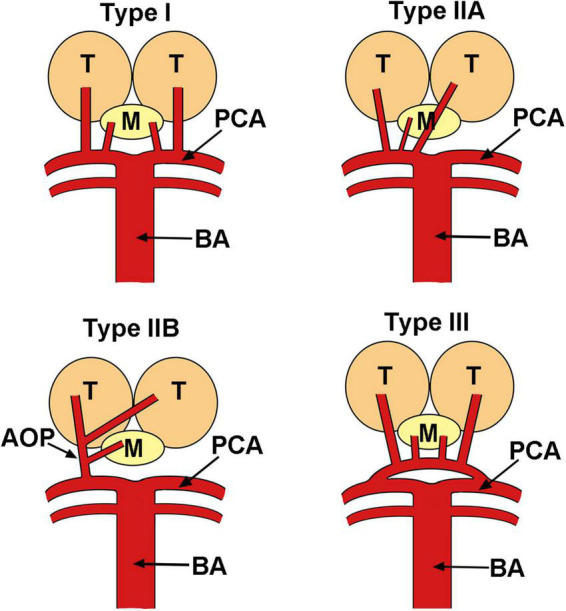
Variants of the vascular anatomy of the thalami and the midbrain. AOP, artery of Percheron; BA, basilar artery; M, midbrain; PCA, posterior cerebral artery; T, thalamus.

The artery of Percheron is a rare anatomic variant that provides arterial supply to the paramedian thalami and rostral midbrain, which was first described by Gérard Percheron in 1973 (7). It is a single arterial trunk that arises from the posterior cerebral artery, which significantly differs from other variants of the thalamic vascular supply (Figure 4). The incidence of AOP was reported to be 4–12% of the population (16), and AOP infarction only counts for 0.7% of all ischemic strokes (5). The main cause of AOP infarction was cardioembolism and small artery diseases (5, 8). The clinical manifestations of AOP infarction are extremely variable, including bilateral vertical gaze palsy, memory impairment, coma, and hypersomnolence (16). Altered consciousness, dysarthria, and motor paresis may indicate ischemia of posterior circulation territory. Memory loss, cognitive impairment, and executive could last months after the first episode. Behavioral consequences including apathy, inappropriate social conduct, and flattened emotions were reported (17). In our reported case, severe drowsiness was noted as the main presentation of AOP infarction. This altered mental status could be explained by the interruption of dopaminergic and noradrenergic impulses from the ascending reticular activating system to the thalamus (16).

The heterogeneity and complexity of AOP infarction explain why it is always lately diagnosed or even not detected. Apart from clues from manifestations, the imaging test plays an important role in its diagnosis. Currently, MRI is the gold standard to demonstrate AOP infarction (9), and routine initial CT scan is often normal (6, 9, 16). Lazzaro et al. first identified four patterns of AOP infarction, including bilateral paramedian thalamic with midbrain (43%), bilateral paramedian thalamic without midbrain (38%), bilateral paramedian thalamic with anterior thalamus and midbrain (14%), and bilateral paramedian thalamic with anterior thalamus without midbrain (5%) (18). Along with the “V” sign on FLAIR and DWI sequences, these ischemic patterns enable the quick diagnosis of AOP infarction.

Apart from AOP infarction, several other diseases could also lead to bilateral thalamic lesions (19, 20). Top of the basilar syndrome may occasionally result in infarcts in posterior cerebral artery territories but often alone with the superior cerebellar. Deep venous thrombosis may result in bilateral symmetric involvement of the thalami. While abnormally hyperdense veins could be seen on CT scans, corresponding T1 hyperintensity from a clot in the sinuses may be seen on MR images (6, 20, 21). Other differential diagnoses could include primary neoplasm, metabolic and toxic disorders, and infections (20).

As with other ischemic strokes, the most effective treatment for AOP infarction is intravenous heparin and thrombolysis with a tissue plasminogen activator, especially for those within the therapeutic window (6 h) (8). However, due to the usually delayed diagnosis, seldom could thrombolysis be carried out in clinic practice (22). In our case, AOP infarction was confirmed on the third day since hypersomnolence, which has been out of the thrombolysis window. An oral anticoagulant was prescribed to prevent future episodes. The patient’s mental status gradually improved on the tenth day. This may attribute to discontinued anticoagulation therapy and unaffected midbrain arterial supply. As reported, even without therapy, the prognosis is significantly better when the midbrain is unaffected, which counts for 43% of AOP occlusion cases (8).

In summary, we reported one case of AOP infarction after an AF ablation procedure whose main manifestation was severe drowsiness. Initial CT was normal, and lately performed MRI confirmed the diagnosis. Due to being out of the thrombolysis window, oral anticoagulant, supporting therapy, and rehabilitation were performed. The patient’s condition improved and was discharged on the tenth day, and the 6-month follow-up confirmed the full recovery. Further systematic clinical studies are needed to further clarify the early diagnosis and optimal management of AOP infarction. In addition, attention should be paid to cardiologists performing catheter ablation when facing patients with atypical altered mental status during the perioperative period.

## Data availability statement

The original contributions presented in this study are included in the article/Supplementary material, further inquiries can be directed to the corresponding author.

## Ethics statement

The studies involving human participants were reviewed and approved by the Institutional Review Board of Shanghai East Hospital. The patients/participants provided their written informed consent to participate in this case study. Written informed consent was obtained from the individual(s) for the publication of any potentially identifiable images or data included in this article.

## Author contributions

XX did the writing work on manuscript. XW, JY, XZ, YW, ZC, JZ, and BZ collected the data. XL and BY guided the whole progress of the presentation. LS did the drawing work. All authors have read and approved the manuscript.

## Supplementary material

Supplementary Figure 1 Timeline of events during the hospital stay. AF, atrial fibrillation; CT, computed tomography; CCU, cardiac care unit; AOP, artery of Percheron.
