# Supplementary figures and images for: Case report: Artery of Percheron infarction as a rare complication during atrial fibrillation ablation
Source: Front Cardiovasc Med. 2022 Sep 13;9:914123. doi: 10.3389/fcvm.2022.914123 (PMC9513031; doi:10.3389/fcvm.2022.914123)

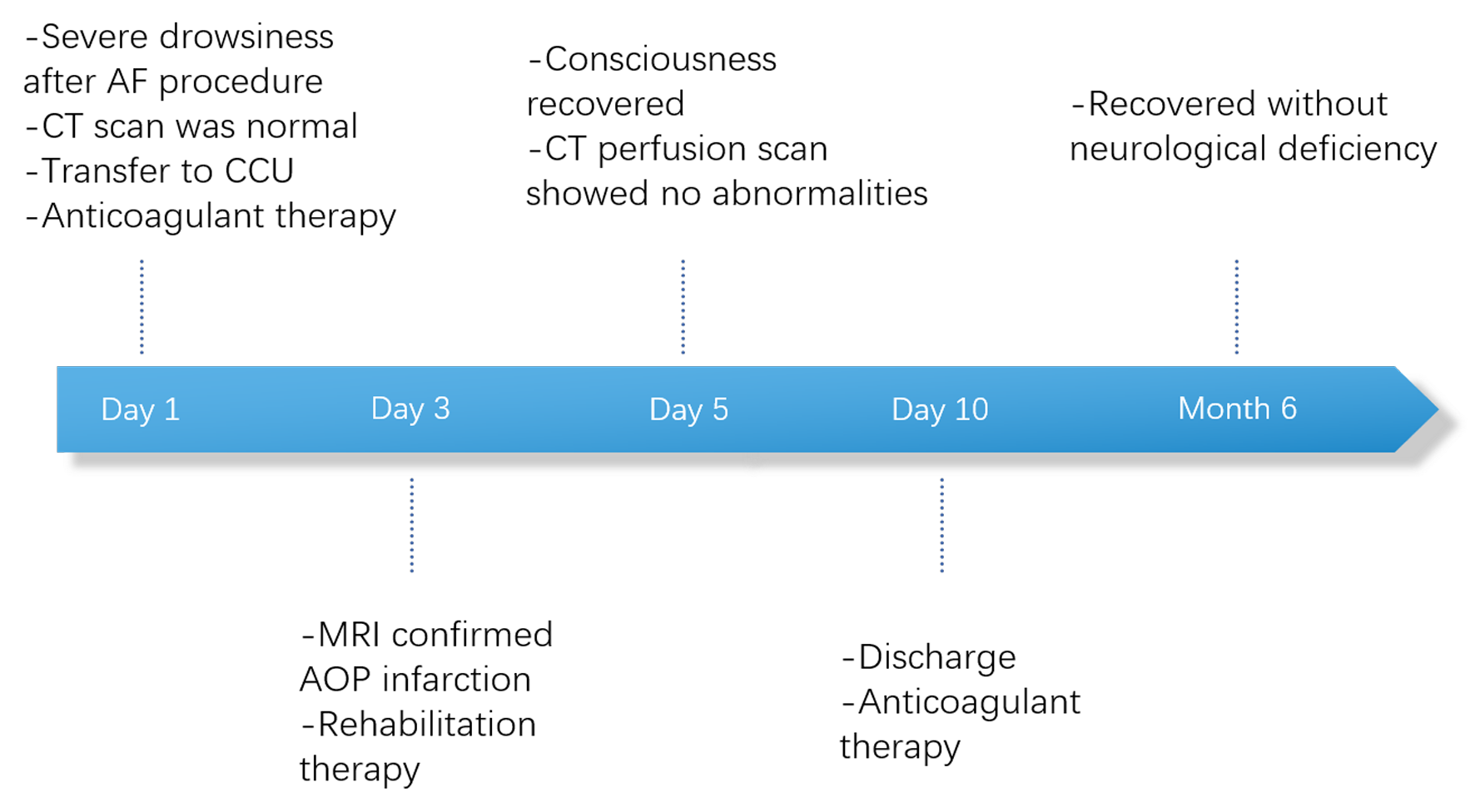

Supplement: Supplementary Figure 1 — Timeline of events during the hospital stay. AF, atrial fibrillation; CT, computed tomography; CCU, cardiac care unit; AOP, artery of Percheron. [file Image_1.TIF]
